# Bundled care in acute kidney injury in critically ill patients, a before-after educational intervention study

**DOI:** 10.1186/s12882-020-02029-8

**Published:** 2020-09-03

**Authors:** Jacqueline Koeze, Iwan C. C. van der Horst, Renske Wiersema, Frederik Keus, Willem Dieperink, Eline G. M. Cox, Jan G. Zijlstra, Matijs van Meurs

**Affiliations:** Department of Critical Care, University of Groningen, University Medical Center Groningen, Postbus 30.001, 9700 RB Groningen, The Netherlands

**Keywords:** AKI prevention, ICU, Care-bundle

## Abstract

**Background:**

Acute kidney injury (AKI) often occurs in critically ill patients. AKI is associated with mortality and morbidity. Interventions focusing on the reduction of AKI are suggested by the Kidney Disease: Improving Global Outcomes guideline. We hypothesized that these educational interventions would improve outcome in patients admitted to the Intensive Care Unit (ICU).

**Methods:**

This was a pragmatic single-centre prospective observational before-after study design in an ICU in a tertiary referral hospital. All consecutive patients admitted to the ICU irrespective their illness were included. A ‘Save the Kidney’ (STK) bundle was encouraged via an educational intervention targeting health care providers. The educational STK bundle consisted of optimizing the fluid balance (based on urine output, serum lactate levels and/or central venous oxygen saturation), discontinuation of diuretics, maintaining a mean arterial pressure of at least 65 mmHg with the potential use of vasopressors and critical evaluation of the indication and dose of nephrotoxic drugs. The primary outcome was the composite of mortality, renal replacement therapy (RRT), and progression of AKI. Secondary outcomes were the components of the composite outcome the severity of AKI, ICU length of stay and in-hospital mortality.

**Main results:**

The primary outcome occurred in 451 patients (33%) in the STK group versus 375 patients (29%) in the usual care group, relative risk (RR) 1.16, 95% confidence interval (CI) 1.03–1.3, *p* < 0.001. Secondary outcomes were, ICU mortality in 6.8% versus 5.6%, (RR 1.22, 95% CI 0.90–1.64, *p* = 0.068), RRT in 1.6% versus 3.6% (RR 0.46, 95% CI 0.28–0.76, *p* = 0.002), and AKI progression in 28% versus 24% (RR 1.18, 95% CI 1.04–1.35, *p* = 0.001).

**Conclusions:**

Providing education to uniformly apply an AKI care bundle, without measurement of the implementation in a non-selected ICU population, targeted at prevention of AKI progression was not beneficial.

## Background

Acute kidney injury (AKI) occurs in 15 to 33% of all critically ill patients [1, 2]. The initial definition of AKI was defined as an increase in serum creatinine and/or a reduction in urine output by Bellomo et al. in 2004, which was revised by Mehta et al. in 2007 and once again in the current definition by Kidney Disease: Improving Global Outcomes (KDIGO) in 2012 [3–5]. Irrespective of the definition, the occurrence of AKI is associated with increased mortality rates and also increased incidences of chronic kidney disease (CKD) after discharge [6, 7]. The severity of AKI is associated with increased intensive care unit (ICU) and hospital mortality [8].

Despite the lack of evidence for benefit, guidelines directed to prevent AKI recommend either volume resuscitation or volume restriction, promote and dismiss various types of fluids, pose blood pressure targets, and emphasize avoidance of nephrotoxic drugs [5]. The recommendation of controlled fluid resuscitation is based on observations that both hypovolemia and hypervolemia can induce AKI [9]. The use of artificial colloids and (loop-) diuretics is dissuaded based on aggregated data from randomised trials and large cohort studies [5, 10]. Nephrotoxic drugs and drug dosing should be weighed carefully, especially in critically ill patients already at risk for AKI [5]. Further, guidelines recommend targeting blood pressure at a mean arterial pressure (MAP) above 60–65 mmHg using vasopressors [5, 11].

A bundled approach of care for the critically ill including these individual recommendations is suggested to preserve renal function [5]. Care bundles targeted at the treatment of sepsis and ventilator-associated pneumonias (VAP) have improved outcomes in critically ill patients [12–14]. Bundles targeted at AKI prevention have already been successfully tested in patients undergoing cardiac surgery and also in hospitalized patients, but not in a general critically ill population [15, 16].

We therefore hypothesized that the implementation of education for bundled care targeted at prevention of AKI progression and a reduction in AKI severity, would improve patient outcome.

## Methods

### Study design

A pragmatic single-centre prospective observational before-after study design was used. The study was performed in a tertiary referral hospital. The need for informed consent was waived by the Institutional Review Board of our hospital (METc 2013–174). The Ethical Board waived consent because the care bundle was implemented at a department level and changed overall daily practice. Patients were treated according to usual care during the first period. After the first (‘before’) period of the study the educational intervention was introduced at a department level to all personnel between June 12th, 2015 and October 14th, 2015. During the second period patients were treated according to the ‘Save the Kidney’ (STK)-bundle in addition to usual care. The duration of both study periods was estimated from admittance rates combined with sample size calculations.

### Patient selection

Consecutive patients admitted to the ICU were included irrespective their illness. Both scheduled post-surgery patients needing postoperative ICU observation and acutely admitted patients, both surgical and medical, were included. Patients with known chronic kidney disease (defined by a serum creatinine greater than 177 μmol/l, based on the definition used by the Nationale Intensive Care Evaluatie (NICE)) and patients on chronic renal replacement therapy (RRT) were excluded from the analysis. If patients were readmitted to our ICU within the study period only data of their first admission were included in the analysis. Patients were splitted into two groups based on admission date, divided into two set periods of time.

### Data collection

Baseline data of all patients were recorded, including age, sex, body mass index (BMI), APACHE IV and admission category and type (medical or surgical; scheduled or emergency). In addition to age and severity of illness, the presence of known diabetes mellitus was recorded as it is a known risk factor for AKI development. We recorded mortality (ICU and hospital), the need for RRT, the occurrence and severity of AKI and the length of ICU stay. Serum creatinine was recorded each day and urinary output was recorded hourly. The reference creatinine was based on the ideal serum creatinine, which was calculated assuming a clearance of 75 ml/min/1.73m^2^ using the ‘modification of diet in renal disease’ (MDRD) formula. The incidence and severity of AKI were assessed in each patient according to the KDIGO definitions, using both serum and urine output criteria (Supplementary figure 1). AKI progression was defined as any progression (i.e. 0 to 1, 2 to 3, etc.) in AKI stage during the first 48 h of admission.

### The ‘save the kidney’ educational intervention bundle

The STK bundle was encouraged in all patients (Table 1). The STK bundle consisted of optimizing the fluid balance (based on urine output, serum lactate levels and/or central venous oxygen saturation), discontinuation of diuretics, maintaining a MAP of at least 65 mmHg with the potential use of vasopressors and critical evaluation of the indication and dose of nephrotoxic drugs [5]. Discouraging artificial colloids was not part of the bundle, since colloids were already excluded in the past (Table 1). Avoidance of hyperglycaemia as is suggested by the KDIGO guidelines was not part of the bundle, as we already used a computerised algorithm regulating serum glucose which was the same in both groups [17]. Close monitoring of serum creatinine, also suggested by the KDIGO guidelines, was also the same in both groups. As mentioned before we measured serum creatinine daily and urine output hourly.

**Table 1:** The ‘Save the Kidney’-bundle

Optimize fluid balance	CVP 8–12 mmHg
	UO > 0.5 ml/kg/h
	serum lactate < 2 mmol/l
	SvO2 > 65%
Stop diuretics	
MAP > 65 mmHg	
Evaluate the indication and dose of possible nephrotoxic medication	

*CVP* Central venous pressure, *UO* Urinary output, *SvO2* Mixed venous oxygen saturation, *MAP* Mean arterial pressure

The ‘STK’-bundle was introduced using introductory lectures to all physicians and nurses, by the distribution of pocket cards, and emphasized during discussions on the wards. The specific interventions were left open for the treating physician to make the bundle more compliant with different personal preferences within the treating intensivist group. We did not assess compliance with the bundle.

### Outcomes

The primary outcome measure was a composite of serious adverse events (SAE’s) consisting of ICU mortality, the need for RRT and/or the progression of AKI.

Secondary outcomes were the three individual components of the composite primary outcome, including ICU mortality, the need for RRT, and the progression of AKI during the first 48 h of ICU stay. In-hospital mortality and ICU length of stay were considered as well.

### Sample size estimation

A previous study in a comparable critically ill population observed a 30-day mortality of 17%, a cumulative incidence proportion of approximately 5% for RRT and the cumulative incidence proportion of 16% for AKI [1]. Based on these data, we estimated a cumulative risk of 25% of one or more of the SAE in the control group of our ICU population. With a cumulative event rate of 25% in the control group, a relative risk reduction (RRR) of 20% of SAE (which would imply a reduction of SAE from 25 to 20%, which was considered clinically relevant), a type I error of 0.05, and a type II error of 0.20, we calculated that 1094 patients were needed in each group of the study (and 2188 patients in total). We anticipated a study period of 6 months for each group.

### Statistical analysis

The proportions of AKI were calculated based on data of the first 48 h of ICU admission. AKI severity was calculated in all patients. Dichotomous data were presented as percentages. Continuous variables were reported as means (with standard deviations (SD)) or as medians (with interquartile ranges (IQR)) depending on normality. Data were analysed using Student t-tests, Mann Whitney U tests, or Chi-square tests, when appropriate. As a sensitivity analysis, analysis were repeated in patients with and without diabetes mellitus.

Missing hourly urine output data were replaced based on averages using the first recorded value over the missing hours. Urine output data were omitted from the analysis if all hourly urine output was missing.

## Results

**Fig. 1:**
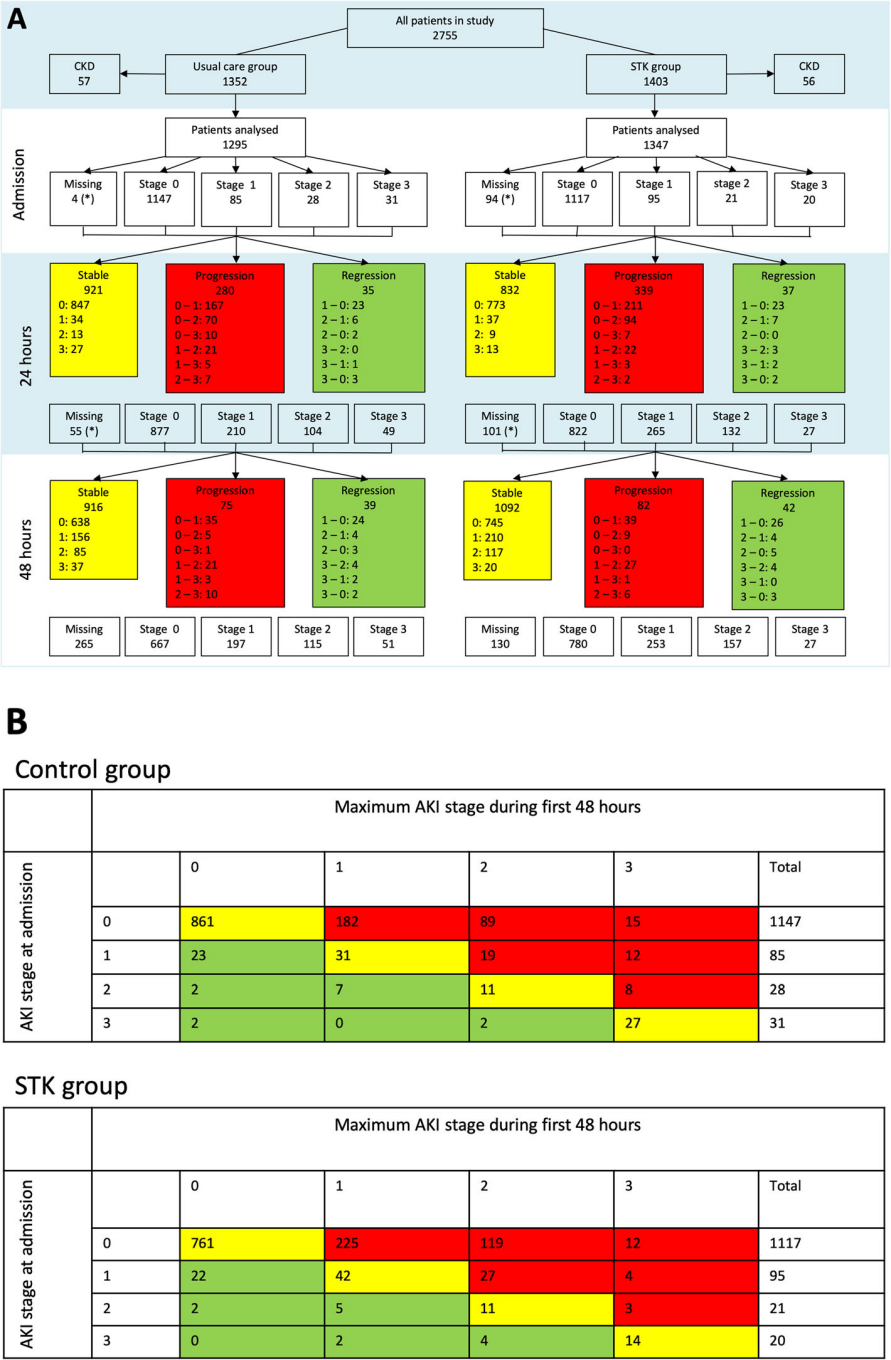
Flow diagram of patients in the study. **a**. Numbers of patients admitted in both groups. Numbers of patients excluded due to presence of chronic kidney disease (CKD). Numbers of patients with severity of acute kidney injury (AKI) and with stable AKI, AKI progression or regression at admission, after 24 h of admission and after 48 h of admission. (*) Usual care group: 4 missing AKI stage at admission, 2 stage 0 after 24 h and 2 stage 1 after 24 h. Save the kidney group: 94 missing AKI stage at admission, 24 stage 0 at 24 h, 8 stage 1 at 24 h, 4 stage 2 at 24 h, 2 stage 3 at 24 h and 56 missing AKI stage after 24 h. Usual care group: 55 missing AKI stage at 24 h all remain missing after 48 h. Save the kidney group: 101 missing AKI stage at 24 h, 1 is stage 0 after 48 h, 100 are missing AKI stage at 48 h. **b**. Number of patients in the control group and STK group with severity of AKI at admission and development over the first 48 h. Green squares indicate improvement in AKI during the first 48 h . Yellow squares indicate stable AKI during the first 48 h. Red squares indicate AKI progression during the first 48 h

A total of 3822 patients were admitted between the start of the study January 1st, 2014 and study closure on March 15th, 2015. The first period was from 01 to 01-2014 until 11-06-2014 and the second period was from 15 to 10-2014 until 15-03-2015. A total of 1295 patients (49%) were included in the usual care group and 1347 patients (51%) were included in the STK group. (Fig. 1a).

### Baseline patient characteristics

Patients in both groups were similar regarding age, sex, BMI, severity of illness and the presence of known diabetes mellitus (Table 2). More patients in the STK group were admitted for a medical reason and less patients were admitted after scheduled surgery (Table 2). Patients in the STK group had a lower median cumulative fluid balance during the first 3 days of ICU admission.

**Table 2:** Basic patient characteristics

Characteristic	Usual care group	STK group	*P*-value
Number of patients	1295	1347	
Age (years; mean ± SD)	60.2 (15.6)	59.6 (16.2)	0.35
Male sex (N (%))	809 (63)	835 (62)	0.8
Body Mass Index (kg/m2; mean ± SD)	26.5 (5.2)	26.5 (10)	0.98
APACHE IV (mean ± SD)	51 (25)	52.3 (25)	0.18
Admission type			0.05
● Medical (N (%))	423 (33)	497 (37)	
● Surgical (N (%))			
○ Scheduled (N (%))	699 (54)	665 (49)	
○ Emergency (N (%))	172 (13)	185 (14)	
Co-morbidity			
● Diabetes mellitus (N (%))	194 (15)	195 (15)	0.72
Serum creatinine at admission (μmol/l; median (IQR))	73 (59–91)	72 (59–89)	0.16
KDIGO stage admission (N (%))	1291 (99)	1253 (93)	0.73
No AKI (N (%))	1147 (89)	1117 (83)	
Stage 1 (N (%))	85 (6.6)	95 (7.1)	
Stage 2 (N (%))	28 (2.2)	21 (1.6)	
Stage 3 (N (%))	31 (2.4)	20 (1.5)	
Fluid balance day 1	1086 (299, 2112)	728 (61, 1795)	< 0.01
Fluid balance day 2	848 (194, 2260)	726 (173, 1878)	0.023
Fluid balance day 3	708 (89, 1785)	533 (−13, 1389)	0.011

*STK* ‘Save the Kidney’, *SD* Standard deviation, *APACHE* Acute physiology and chronic health evaluation, *IQR* Inter quartile range

Serum creatinine at admission was unavailable in 98 patients (3.7%) and in 489 patients (19%) there was insufficient data to estimate the presence or absence of AKI based on urine output criteria on admission. Overall, the median available hours of urine output data calculated as a percentage of length of stay was 89% (IQR 62–95%).

### Primary outcome

Serious adverse events defined as ICU mortality, the need for RRT and/or the progression of AKI was observed in 451 patients (33%) in the STK group compared to 375 patients (29%) in the usual care group (RR 1.16, 95% confidence interval CI 1.03–1.3, *p* < 0.001) (Table 3). The AKI progression during the first 48 h after ICU admission was not different between the STK group and the usual care group (Table 3 and Fig. 1b).

**Table 3:** Primary and secondary outcomes of the ‘Save the Kidney’-bundle

Outcome	Usual care group	STK group	RR (95%CI) *P*-value
Number of patients	1295	1347	
Primary outcome (AKI progression, need for RRT and ICU mortality) (N (%))	388 (30)	465 (36)	1.15 (1.03–1.29)
			0.001 (*)
Overall ICU mortality (N (%))	72 (5.6)	91 (6.8)	1.22 (0.90–1.64)
			0.068 (*)
Need for RRT during ICU admission (N (%))	46 (3.6)	22 (1.6)	0.46 (0.28–0.76)
			0.002 (*)
AKI progression (N (%))	326 (25)	402 (31)	1.19 (1.05–1.34)
			0.001 (**)
Maximum AKI stage during 48 h			0.87 (0.82–0.93)
No AKI (N (%))	863(67)	783 (61)	< 0.001 (***)
AKI stage 1 (N (%))	238 (18)	299 (23)	1.21 (1.04–1.41)
AKI stage 2 (N (%))	128 (10)	169 (13)	1.27 (1.02–1.58)
AKI stage 3 (N (%))	66 (5.1)	41 (3.2)	0.6 (0.41–0.88)
ICU LoS (days; median [IQR])	2 [2–3]	2 [2–3]	0.319 (*)
Hospital mortality (N (%))	117 (9)	137 (10.2)	0.248 (*)

*STK* ‘Save the Kidney’, *AKI* Acute kidney injury, *RRT* Renal replacement therapy, *ICU* Intensive care unit, *LoS* Length of stay, *IQR* Inter quartile range, *RR* Relative risk, *CI* Confidence interval. (*): *p* value considering difference between outcome in the usual care group and the STK group. (**) *p* value considering difference in AKI incidence between both groups. (***) *p* value considering difference in AKI severity between both groups

### Secondary outcomes

#### ICU mortality

In the STK group 91 patients (6.8%) died during their ICU admission compared to 72 patients (5.6%) in the usual care group (RR 1.22, 95% confidence interval 0.90–1.64, *p* = 0.068) (Table 3).

#### Need for RRT

RRT was used in 22 patients (1.6%) in the STK group versus 46 patients (3.6%) in the usual care group RRT (RR 0.46, 95% confidence interval 0.28–0.76, *p* = 0.002) (Table 3).

#### AKI progression

Based on serum creatinine and urine output criteria 383 patients (28%) in the STK group developed AKI progression versus 311 patients (24%) in the usual care group (RR 1.18, 95% confidence interval 1.04–1.35, *p* = 0.001).

AKI progression based on the separate components serum creatinine and urine output is shown as supplementary material (supplementary data 1 and supplementary table 1). The sensitivity analysis showed that patients with diabetes mellitus did not show a difference in outcome (supplementary table 2).

#### AKI severity

Based on serum creatinine and urine output AKI severity differed in the STK group compared with the usual care group. In the STK group 299 patients (23%) had stage 1 AKI and in the usual care group 238 patients (18%). In the STK group 169 patients (13%) had stage 2 AKI and in the usual care group 128 patients (10%). In the STK group 41 patients (3.2%) had stage 3 AKI and in the usual care group 66 (5.1%) (*p* < 0.001) (Table 3).

AKI severity based on the separate components serum creatinine and urine output is shown in the supplementary table 1.

#### ICU length of stay

Median ICU length of stay in the STK group was 2 days [IQR 2–3] and in the usual care group also 2 days [IQR 2–3] (*p* = 0.319) (Table 3).

#### In-hospital mortality

In the STK group 137 patients (10%) died during their hospitalisation and in the usual care group 117 patients (9%) (RR 1.13, 95% confidence interval 0.89–1.42, *p* = 0.248) (Table 3).

## Discussion

We used a pragmatic before-after design to test whether a bundled approach targeted at prevention of AKI and a reduction of AKI severity and AKI progression, introduced at a department level impacts the outcome of critically ill patients. This study showed that implementation of an educational ‘Save the Kidney bundle care in critically ill patients aiming at a reduction of AKI had no beneficial effect on patient outcome when evaluated by a composite of ICU mortality, the need for RRT and AKI progression.

These results are in contrast with the few studies that evaluated AKI care bundles in hospitalized patients in general or in patients after cardiac surgery [15, 16]. Those studies showed either a reduction in AKI incidence and AKI severity in cardiac surgery patients or a reduction in AKI incidence and a reduction in-hospital mortality. Our study was not powered to detect significance in individual components of the primary endpoint. The primary outcome showed no benefit as a result of a higher AKI incidence and contrary, a lower need for RRT in the STK group (Table 3). In our study ICU mortality was higher in the STK group, albeit non-significant. Therewith, the effects of the implemented bundle were contradictory, given the fact that need for RRT is associated with a higher mortality.

Our results are also in contrast with other bundles in critically ill patients such as the treatment bundle of the Surviving Sepsis Campaign (SSC) to reduce mortality in patients with sepsis or the bundle to reduce incidence of ventilator associated pneumonia (VAP) in mechanically ventilated critically ill patients [13, 14]. It is important to note however that our study comprised an educational intervention of which affects are often difficult to assess.

### Strengths and limitations

Our study has several limitations. First, we did not record which patients had the bundle applied in their treatment. Besides that, we did not record which part of the bundle was applied in which patients. It is possible that one of the suggested interventions of the bundle has detrimental effects on the composite primary outcome, while others may have positive effects. Moreover, not all interventions were clearly described, but adjustments in antibiotic dose were based on renal clearance and accordingly appropriate drug dosing. This also holds for the optimization of the fluid balance, physicians and nurses were stimulated to critically evaluate the need for fluids. This was based on the formulation of the KDIGO guidelines.

Second, we did not assess the full MAKE30 endpoint, which was not published at the time. However, our primary endpoint was the composite of death, new renal replacement therapy, or persistent renal dysfunction which is recommended, as a patient-centered outcome for pragmatic trials involving AKI.

Third, we did not analyse the compliance of the bundle and furthermore, the same bundle can be implemented differently by different clinicians. A recent study showed that using an interruptive AKI alert therapeutically interventions in patients increased from 7.9 to 28.7% of the patients [18]. Therefore, it is likely that the compliance of this study was not 100%. Future research of our group and others on the effect of AKI care bundles in critically ill patients need to address this issue.

Fourth, we did not correct for all previously reported comorbidities, hence, differences in illness severity between groups may have played a role in our findings. Furthermore, we had insufficient urine output data to estimate AKI incidence and severity based on urine criteria in nearly 20% of patients, with unequal amounts of missing data between the two groups. This might have led to underestimation of AKI incidence in the usual care group since urine output is more sensitive in detecting AKI than serum creatinine. This could have exaggerated the (negative) effect of the care bundle. Last, we did not collect baseline creatinine but estimated renal function based on the assumption of a glomerular filtration rate of 75 l/min/1.73 m^2^.

A strength of this study is the prospective study design with the inclusion of all patients, except for patients with CKD, which reflects real life daily practice and patient population. Even though this resulted in relatively low median APACHE scores, AKI incidence was still high and the KDIGO guidelines suggest that the measures they advise may reduce AKI incidence in general.

No comparable studies have been performed in critically ill patients. We constructed an educational intervention regarding bundled elements of AKI care as advised by international guidelines. Each of the measures is supported by literature and guidelines suggest beneficial effects of bundled care. The results of our study show a contradictory effect. This may be caused by limitations of the current study or by chance, but it may also be the result of the bundle itself. The simultaneously taken measures rather than measures taken subsequently might induce harm. A possible cause is for example the reduced fluid resuscitation with possible adverse effects on outcome due to – relative – hypovolemia. Also, the reductions in antibiotic dosing propagated by the bundle may have led to insufficient treatment of infections. This may support therapeutic drug monitoring (TDM) [19]. In future research protocol adherence and effects of implementation need to be studied.

## Conclusion

Although more patients in the control group developed stage 3 AKI compared to those in the STK intervention group, providing education to uniformly apply an AKI care bundle, without measurement of the implementation in a non-selected ICU population, targeted at prevention of AKI progression was not beneficial.

## Supplementary information

**Additional file 1: Figure S1.** AKI classification according to the KDIGO guideline.

**Additional file 2.** Supplementary data 1.

**Additional file 3: Table S1.**

**Additional file 4: Table S2.**

## Data Availability

Please contact the author for data requests.

## Abbreviations

AKI: Acute kidney injury
BMI: Body Mass Index
CKD: Chronic Kidney Disease
ICU: Intensive Care Unit
IQR: Inter Quartile Range
KDIGO: Kidney Disease Improving Global Outcome
MAP: Mean Arterial Pressure
MDRD: Modification of Diet in Renal Disease
NICE: Nationale Intensive Care Evaluatie
RR(R): Relative Risk (Reduction)
RRT: Renal Replacement Therapy
SAE: Serious Adverse Event
SSC: Surviving Sepsis Campaign
SD: Standard Deviation
STK: Save The Kidney
VAP: Ventilator-associated Pneumonia
