# Supplementary material for: Bundled care in acute kidney injury in critically ill patients, a before-after educational intervention study
Source: BMC Nephrol. 2020 Sep 3;21:381. doi: 10.1186/s12882-020-02029-8 (PMC7469422; doi:10.1186/s12882-020-02029-8)
Supplement: Supplementary file 1 — Additional file 1: Figure S1. AKI classification according to the KDIGO guideline. [file 12882_2020_2029_MOESM1_ESM.docx]

Supplementary figure 1. AKI classification according to the KDIGO guideline

|  | **serum creatinine criteria** | **urine output criteria** |
| --- | --- | --- |
| stage 1 | creatinine *1.5-1.9  or  creatinine + ≥ 0.3 mg/dl (26.5 µmol/l) | <0.5 ml/kg/h ≥ 6h |
| stage 2 | creatinine * 2.0-2.9 | <0.5 ml/kg/h ≥ 12h |
| stage 3 | creatinine *3  or  creatinine ≥ 4 mg/dl (354 µmol/l)  or  RRT | < 0.3 ml/kg/h ≥ 24h  or  Anuria ≥ 12h |

RRT = renal replacement therapy
