# Supplementary material for: Bundled care in acute kidney injury in critically ill patients, a before-after educational intervention study
Source: BMC Nephrol. 2020 Sep 3;21:381. doi: 10.1186/s12882-020-02029-8 (PMC7469422; doi:10.1186/s12882-020-02029-8)
Supplement: Supplementary file 2 — Additional file 2. Supplementary data 1. [file 12882_2020_2029_MOESM2_ESM.docx]

Supplementary data 1

*AKI progression*

Based on serum creatinine criteria alone 84 patients (6.6%) in the STK group developed AKI during admission versus 98 patients (7.6%) in the usual care group (RR 0.82, 95% confidence interval 0.62 – 1.09). (Supplemental table 2)

Based on urine output criteria alone 414 patients (34%) in the STK group developed AKI versus 332 patients (35%) in the usual care group (RR 1.2, 95% confidence interval 1.07 – 1.36). (Supplementary table 1)

*AKI severity*

Based on serum creatinine and urine output alone AKI severity was not significantly different between the STK group and the usual care group. (p = 0.33 and p = 0.72 respectively) (Supplementary table 1)
