# Supplementary material for: Bundled care in acute kidney injury in critically ill patients, a before-after educational intervention study
Source: BMC Nephrol. 2020 Sep 3;21:381. doi: 10.1186/s12882-020-02029-8 (PMC7469422; doi:10.1186/s12882-020-02029-8)
Supplement: Supplementary file 3 — Additional file 3: Table S1. [file 12882_2020_2029_MOESM3_ESM.docx]

Supplementary table 1.

| Outcome | Usual care group | STK group | RR (95% CI)  p-value |
| --- | --- | --- | --- |
| Number of patients | 1295 | 1347 |  |
| AKI progression based on serum creatinine | 98 (7.6) | 84 (6.6) | 0.82 (0.62 – 1.09)  0.33 (*) |
| Maximum AKI stage  No AKI (N (%)) | 1080 (83) | 1078 (84) | 0.96 (0.93 – 0.99)  0.45 (**) |
| AKI stage 1 (N (%)) | 114 (8.8) | 124 (9.7) | 1.05 (0.82 – 1.33) |
| AKI stage 2 (N (%)) | 43 (3.3) | 44 (3.4) | 0.98 (0.65 – 1.49) |
| AKI stage 3 (N (%)) | 58 (4.5) | 34 (2.7) | 0.56 (0.37 – 0.85) |
| AKI progression based on urine output | 332 (35) | 414 (34) | 1.2 (1.07 – 1.36)  0.72 (*) |
| Maximum AKI stage  No AKI (N (%)) | 615 (65) | 792 (66) | 1.24 (1.15 – 1.33)  0.55 (**) |
| AKI stage 1 (N (%)) | 190 (20) | 248 (21) | 1.25 (1.06 – 1.49) |
| AKI stage 2 (N (%)) | 117 (12) | 154 (13) | 1.27 (1.01 – 1.59) |
| AKI stage 3 (N (%)) | 25 (2.6) | 12 (1.0) | 0.46 (0.23 – 0.91) |

AKI progression and severity in both groups based on serum creatinine and urine output alone.

(*) p-value considering difference in AKI progression between both groups

(**) p-value considering difference in AKI severity between both groups
