# Supplementary material for: Bundled care in acute kidney injury in critically ill patients, a before-after educational intervention study
Source: BMC Nephrol. 2020 Sep 3;21:381. doi: 10.1186/s12882-020-02029-8 (PMC7469422; doi:10.1186/s12882-020-02029-8)
Supplement: Supplementary file 4 — Additional file 4: Table S2. [file 12882_2020_2029_MOESM4_ESM.docx]

Supplementary table 2.

|  | Usual care group | STK group | RR (95% CI) | P value |
| --- | --- | --- | --- | --- |
| Diabetes Mellitus present | | |  |  |
| - AKI progression | 63 | 81 | 1.32  (1.02 – 1.71) | 0.036 |
| - No AKI progression | 131 | 108 |  |  |
| Diabetes Mellitus not present | | |  |  |
| - AKI progression | 293 | 353 | 1.22  (1.07 – 1.39) | 0.003 |
| - No AKI progression | 808 | 736 |  |  |

AKI progression in patients with or without diabetes mellitus.

RR = relative risk, CI = confidence interval
